# Investigations of the Systematics of Crystal Packing Using the Cambridge Structural Database

**DOI:** 10.6028/jres.101.033

**Published:** 1996

**Authors:** Carolyn Pratt Brock

**Affiliations:** Department of Chemistry, University of Kentucky, Lexington, KY 40506-0055

**Keywords:** Cambridge Structural Database, crystal packing, molecular crystals

## Abstract

Several studies that used the Cambridge Structural Database to elucidate principles of packing in molecular crystals are described. Some possible sources of bias in the statistical distributions are discussed.

## 1. Introduction

We have used the Cambridge Structural Database [1] (hereafter, the CSD) to investigate several classical problems of crystal packing in molecular materials. These studies are described briefly below. For a list of the many other publications from other laboratories on similar subjects see the bibliography available within the CSD [1].

## 2. Systematic Effects of Crystal-Packing Forces

Comparisons of geometrical data from large numbers of crystal structures have been used (see [2] for background and a review) to determine mean bond lengths and angles, to identify probable fragment conformations, to infer reaction pathways, and to deduce the nature of transition states. The structure-correlation method [2], on which such studies are based, depends on the solid-state fragment geometries being localized in the low-energy regions of their conformational spaces. The method assumes that the distributions of geometrical parameters over many fragments in different crystal structures will be similar to the distributions over time for a single fragment in a fluid phase.

**Fig. 1:**
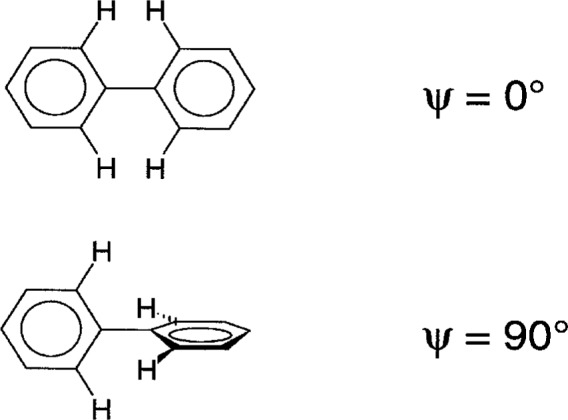
Biphenyl conformations.

But is this assumption always valid? We investigated [3] the distribution of torsion angles *ψ* in biphenyl fragments having H atoms in all four *ortho* positions (see Fig. 1). Biphenyl itself is a classic example of a molecule that has a different conformation in the solid state (*ψ* = 0) than in the gas phase (*ψ* = 44°). The variation in energy with *ψ* was well established both from calculations and from electron-diffraction studies [4]; the energy curve has a minimum at *ψ* ca. 45° and maxima ca. 7 kJ above the minimum at *ψ* = 0° and 90°. If the assumption of randomness in solid-state deformations is valid then the distribution of *ψ* values for biphenyl fragments should have a single maximum at *ψ* ca. 45°. The distribution would be expected to be at least approximately Gaussian.

The *ψ* values obtained from 101 fragments in 68 structures of 65 different molecules (January 1988 version of the CSD) range from *ψ* = 0° to about *ψ* = 60°. The distribution, which is approximately continuous, has maxima at both 0° and 37°. The observed distribution looks nothing like the expected distribution. There are two maxima rather than one. All values *ψ* < 50° are well represented but no structures have *ψ* > 60°.

It appears that crystallization of biphenyls without *ortho* substituents systematically favors “flat” biphenyl conformations (low *ψ* values). The simplest explanation for the observed distribution of *ψ* values is that very twisted biphenyl molecules (*ψ* > 45°) do not fit together very well in the solid state. Nearly planar molecules, however, can pack in the favorable “herringbone” arrangement so often observed for aromatic molecules.

The violation by *ortho* unsubstituted biphenyls of the principle underlying the structure-correlation method is almost certainly an exception, but it does provide a warning. If (1) the overall shape of a fragment can be changed substantially at a small energy cost, and (2) the deformed fragment can be expected to interact more favorably than the undeformed fragment with its neighbors, then the structures retrieved from a database may fail to cluster in the minimum of the isolated-fragment potential energy surface. These conditions, however, are seldom met.

## 3. The Density and Stability of Racemic Crystals Compared With Their Chiral Counterparts

In 1895 Wallach [5] published the observation, which has since been repeated widely (see [6]), that crystals of racemic compounds (1:1 compounds of enantiomers) are denser than their chiral counterparts (enantiomerically pure compounds). This rule was based on eight pairs of densities, one of which was an exception. The difficulty in measuring densities weakened the rule: the differences in densities for each pair were comparable to the errors (ca. 1 %–2 %) in the experimental measurements.

The relatively low frequency (ca. 20 %) of chiral groups in the CSD suggested that Wallach’s rule might indeed be true. It appears (the lack of information on crystallization conditions makes such estimates difficult) that conglomerate crystallization (eutectic deposition of a 1:1 mixture of D and L crystals from a racemic solution or melt) occurs only about one time in nine (see [6]).

Densities determined crystallographically from unit-cell dimensions and compositions are typically known to better than 0.15 %. A comparison of crystallographic densities (or, volume per molecule) for a large number of matched pairs of structures provides a much better test of Wallach’s generalization than was possible before the CSD was available. We therefore decided to make such a comparison [6].

The temperatures of the two structure determinations in a pair had to be the same to within ca. 10 K. It was also important to be sure that no included solvent had been overlooked. We found it necessary to consult the original literature extensively. A total of 129 matched pairs of structures (January 1989 version of the CSD) were located. Compilation of the list of matched pairs of structures was complicated by the lack (in 1989) of any link in the CSD between structures of molecules having the same connectivity and stereochemistry (e.g., l-alanine and dl-alanine).

It became clear that the pairs had to be separated into two groups. Group I includes all achiral substances (e.g., glycine) and all molecules (or ions) whose enantiomers interconvert rapidly (e.g., 1,1′-binaphyl, 4-hydroxybiphenyl). Group II includes all substances that can be resolved (e.g., alanine, heptihelicene). The phase diagrams of these two groups differ thermodynamically: the melt of a group I substance is a one-component system while the melt of a group II substance is a two-component system. Matched pairs of structures of group I substances are polymorphs; matched pairs of structures of a group II substances are different compounds.

Two polymorphs obtained under similar conditions must have similar energies. All group I pairs are composed of polymorphs that have similar energies. The situation for group II substances, however, is very different. The energies of crystals of enantiomerically pure material and of the racemic compound may be comparable or they may not. Even if the racemic compound is much more stable, crystals of the homochiral material can still be grown from solutions or a melt of enantiomerically pure (i.e., resolved) material. The list of matched pairs *cannot*, however, include any pair for which crystals of enantiomerically pure material are much more stable than crystals of the racemic compound. If crystals of the racemic compound are much less stable than crystals of enantiomerically pure material the compound simply disappears from the (equilibrium) phase diagram. There is no way to force crystallization of a racemic compound that is unstable relative to the corresponding conglomerate.

A total of 64 pairs of structures of group I substances were found. The difference in densities $(\Delta \left(\%\right)=100\;\Delta \rho /\left(\frac{1}{2}\Sigma \rho \right)$, where *ρ* is the compound density) was 0.20(34) %, i.e., essentially zero. The distribution was reasonably symmetric about *Δ* (%) = 0. This result supports the expectation that structures having similar energies should have similar densities.

The value of *Δ* (%) for the 65 pairs of group II structures was 0.92(29) %. On average racemic compounds are about 3.2*σ* denser than crystals of the corresponding enantiomerically pure material. Wallach’s observation is valid. The significance of his observation, however, is less clear. Matched pairs of structures can be obtained only if crystals of the racemic compound are at least as stable as crystals of the enantiomerically pure material, and the more stable crystal is expected to be, at least on average, more dense. The absence of pairs for which the racemic compound is less stable means that the comparison includes a systematic bias that cannot be eliminated. Hence *no* such tabulation (density, *T*_fus_, *Δ*H_fus_, *Δ*S_fus_, etc.) of matched pairs should be used to support the assertion that crystals of a racemic compounds can be expected to be more stable than crystals of the corresponding homochiral material. The low frequency of chiral groups in the CSD, however, leaves open the possibility that Wallach’s observation, although based on a flawed comparison, is, nevertheless, true.

## 4. Space-Group Frequencies

The prediction of the crystal structures for a molecule of known geometry remains an elusive goal, even if the molecular surface is simple. The crystallographic databases have made possible, however, the discovery of generalizations about crystal packing. One approach is to examine the relative frequencies of the different space groups. Papers on this subject first appeared in the 1940s; for a review see [7]. The role of imposed molecular (or ionic) symmetry had, however, been largely neglected. We undertook [7] to generate new tables of space-group frequencies (January 1991 version of the CSD) in which the variable *Z′* (the number of formula units in the asymmetric unit) was included explicitly. Wilson [8] produced a similar set of tables at about the same time.

The new tables [7, 8] show that space groups having mirror symmetry do not occur unless molecules or ions are located on *all* the mirror planes. Groups with 3-, 4-, and 6-fold rotation axes seldom occur unless the molecules or ions are located on the axes. Twofold rotation axes are sometimes occupied and sometimes not. Points of inversion symmetry are very often *un*occupied. There is seldom more than one molecule in the asymmetric unit unless the overall symmetry is especially low, in which case structures with *Z′* > 1 are quite common. Overall the average number of molecules in the unit cell is surprisingly constant over the crystal systems, even though the number of equivalent positions in the cell increases rapidly as symmetry elements are added.

These tables made possible some observations and predictions about crystal packing. A possible strategy (see also [9]) for the preparation of crystals with nonlinear optical properties is to use molecules and/or ions likely to crystallize in high-symmetry systems (trigonal, tetragonal, hexagonal, cubic) because the commonly occurring groups in these systems lack an inversion center. A comparison of the tables in [7] and [8] shows that high-symmetry groups are more likely for salts and solvates than for crystals containing a single packing unit, presumably because the second fragment breaks up the like-like interactions generated by mirror planes and rotation axes. On the other hand the strategy of using molecules that can conform to twofold rotational symmetry to promote crystallization in noncentrosymmetric groups seems unlikely to succeed. Space group C2/c, which is centrosymmetric, appears to be the most probable group by far (see also [10]) for molecules that retain twofold rotational symmetry in the crystal.

More recent tables of space-group frequencies [10, 11] have identified the fragment symmetry explicitly. In group C2/c, for example, *Z′* = 1 can occur for either inversion or twofold-rotation symmetry. These tables [10, 11] show that the latter symmetry is more common.

The study [7] of space-group frequencies also shed light on Wallach’s rule. Crystallization of racemic compounds almost always [6] occurs in space groups that include symmetry elements of the second kind (inversion centers, mirror and glide planes, improper rotation axes). Crystallization of a racemic compound in a chiral space group is very rare. Mirror planes are unfavorable for crystal packing, glide planes are perhaps neither particularly favorable nor unfavorable, and improper axes (other than $\bar{1}$) do not occur unless molecules are located on the axes. Inversion symmetry, however, is very favorable for crystal packing. Inversion centers prevent like-like interactions without imposing any conditions on the position or orientation of the molecules or ions. The volume of space from which molecules are necessarily excluded by van der Waals interactions is very large for a mirror plane, intermediate for a rotation axis, and smallest for an inversion point. Since higher density is correlated with greater stability, inversion symmetry is better for crystal packing than is rotation or reflection symmetry. Finally, inversion centers are uniquely compatible with translation—the dominant symmetry operation by far in any crystal—because the inversion operation changes the direction, but not the orientation, of the intermolecular vectors. It is therefore not surprising that most structures are centrosymmetric.

## 5. Anomalous Space-Group Frequencies for Monoalcohols, C*_n_*H*_m_*OH

In the course of a routine structure determination of a steroid derivative [12] we encountered a unit cell with a common space group (P2_1_) but a *very* infrequent value [7] of *Z*′ (*Z*′ = 3). The only functional group in the steroid is a single hydroxyl substituent. The –OH groups from the three independent molecules form a helix with pseudo 3_1_ symmetry parallel to *b*.

After thinking about this structure we realized that the space-group distribution for all monoalcohols might be anomalous. Hydroxyl groups are approximately equally good hydrogen-bond donors and acceptors, but seldom (if ever) form closed dimers. Rather, the dominant patterns [13] are chains and rings in which each hydroxyl group interacts with two others. Unless the molecule is very thin the hydroxyl groups from three separate molecules related by translation or by screw axes or glide planes cannot get close enough to form hydrogen bonds. Crystallization in high-symmetry space groups (where 3- and 4-fold screw and rotation-inversion axes are possible) is one solution. Crystallization with *Z*′ ≥ 2 is another.

A search of the October 1992 version of the CSD revealed 55 well determined structures of monoalcohols [14]. There is complete H bonding in only 37 of these; in the remaining 18 there is at least one donor or acceptor that fails to participate (because of steric hindrance) in any O–H⋯O bond.

Of the 37 structures with a complete set of O–H⋯O bonds, 12 (32 %) crystallize in high-symmetry (tetragonal, trigonal, hexagonal, and cubic) space groups. The comparison value for the CSD as a whole is only about 1 %. An additional 19 (51 %) of the C*_n_*H*_m_*OH molecules crystallize with *Z*′ ≥ 2. The comparison value for the CSD as a whole is 8 %. Only 6 C*_n_*H*_m_*OH molecules (16 %) crystallize in “normal” space groups with *Z*′ = 1. Three are structures of “thin” molecules that can be related by 2_1_ axes. The other three C*_n_*H*_m_*OH structures are in space group C2/c, in which molecules can be related by alternating screw axes and glide planes.

This study suggests that hydrogen bonding can be used to favor crystallization in high-symmetry groups, which would also favor (see above) crystallization in noncentrosymmetric space groups. This study also demonstrates that H-bonded aggregates can occupy special positions of symmetry $\bar{3}$ and $\bar{4}$, thus making possible crystallization in trigonal and tetragonal groups (e.g., $R\bar{3}$) even if the molecule has no special symmetry. Consider the recently published structure of a sugar lactone [15], which crystallizes with extensive hydrogen bonding in the cubic group P2_1_3 (#198) with *Z*′ = 1. All of the more than 80 other P2_1_3 structures listed in the March 1995 version of the CSD have *Z*′ = 1/3 (or occasionally 2/3) and have the molecules or ions located on threefold rotational axes.

## 6. Space Groups that Usually Occur Only for Specific Types of Compounds

It is usually assumed that a given space group can accommodate a wide variety of types of molecules and ions. High-symmetry groups with special positions do not occur unless the special positions are occupied [7] but specification of the point-group symmetry places few restrictions on the types of bonds that can be present. Conversely, it is usually assumed that the space group cannot be predicted on the basis of the molecular or ionic symmetry alone. First, location of the molecule or ion on a special position is by no means certain. Even if the molecules or ions lie on special positions there is a selection of groups possible (and actually observed) for most point-group symmetries having rotation axes of order 4 or lower. Racemic and achiral compounds usually crystallize in space groups having improper symmetry operations [6], but that is a very weak restriction.

Space group P4/n (#85) is an exception to these expectations. Of the 84 structures in this group found in the March 1995 version of the CSD, 34 are of the type [MPh_4_]^+^[M′XY_4_]^−^ or [MPh_4_]^+^[M′XYZ_4_]^−^. The high probability that a PPh_4_^+^ or AsPh_4_^+^ salt of a small, simple anion with possible symmetry 4 will crystallize in P4/n was noted by Mueller [16], but the realization that this class of salts dominates the list of structures observed in P4/n is new. The possibility of a favorable arrangement of phenyl groups around an *empty* site of $\bar{4}$ symmetry is key [17]. Of the 84 structures observed in P4/n, 57 have peripheral phenyl groups that surround an empty $\bar{4}$ site.

Half of the ca. 30 structures tabulated [7, 8] for space group P6_3_/m (#176) have the formula [M(H_2_O)_9_]^3+^[C_2_H_5_OSOS_3_]^−^_3_, where M is one of the rare-earth elements. The frequency observed for this space group has been raised by Gerkin and Reppart’s systematic study [18] of 14 very closely related compounds.

## 7. The Databases of All Structures, of Attempted Structures, and of Published Structures

The characteristics of the database of all structures attempted must be different than those of the database of published structures. The difficulty in obtaining a structure solution and a satisfactory refinement for a structure with *Z*′ > 1 means that such structures are almost certainly underrepresented. Disordered, modulated, and incommensurate structures are underrepresented for the same reason. Structures in high-symmetry groups are probably underrepresented because of the difficulties associated with twinning. Tabulations ([7, 8]) show that structures in high-symmetry systems usually crystallize in space groups having the lowest possible Laue symmetry; the probability of merohedral twinning is therefore high. Space-group ambiguities are also a problem, particularly in the tetragonal system.

There is little known about the characteristics of the set of structures of poorly diffracting materials.

Molecules having inversion (i.e., $\bar{1}$) symmetry are overrepresented in the CSD. About 12 % of the structures in the January 1991 version of the CSD have molecules or ions located on inversion centers [7]. Belsky, Zorkaya, and Zorky [10], who used their own database, found that 8 % of the structures had molecules located on an inversion center. These percentages are almost certainly larger than the percentage of molecules listed in Chemical Abstracts that could conform to inversion symmetry. The source of this bias is not clear (see [7]).

## 8. Conclusions

A great deal can be learned about the organization of molecules and ions in crystals from the systematic study of databases. It crucial (as well as informative) to watch out for unexpected sources of bias.

## References

[1] Allen FH, Kennard O, Taylor R (1983). Systematic Analysis of Structural Data as a Research Technique in Organic Chemistry. Acc Chem Res.

[2] Bürgi H-B, Dunitz JD (1994). Structure Correlation.

[3] Brock CP, Minton RP (1989). Systematic Effects of Crystal-Packing Forces: Biphenyl Fragments with H Atoms in All Four Ortho Positions. J Am Chem Soc.

[4] Bastiansen O, Samdal S (1985). Structure and Barrier of Internal Rotation of Biphenyl Derivatives in the Gaseous State. J Mol Struct.

[5] Wallach O (1895). Zur Kenntnis der Terpene und der ätherischen Oele; Vierunddreissigste Abhandlung. Liebigs Ann Chem.

[6] Brock CP, Schweizer WB, Dunitz JD (1991). On the Validity of Wallach’s Rule: On the Density and Stability of Racemic Crystals Compared with their Chiral Counterparts. J Am Chem Soc.

[7] Brock CP, Dunitz JD (1994). Towards a Grammar of Crystal Packing. Chem Mater.

[8] Wilson AJC (1993). Space Groups Rare for Organic Structures. III. Symmorphism and Inherent Molecular Symmetry. Acta Cryst.

[9] Brock CP, Dunitz JD (1994). On the Prevalence of Polar and Chiral Space Groups, Mol. Cryst Liq Cryst.

[10] Belsky VK, Zorkaya ON, Zorky PM (1995). Structural Classes and Space Groups of Organic Homomolecular Crystals: New Statistical Data. Acta Cryst.

[11] Cole JC (1995). Ph D Thesis.

[12] Brock CP, Stoilov I, Watt DS (1994). A Steroid Derivative that Crystallizes with Three Molecules in the Asymmetric Unit. Acta Cryst.

[13] Jeffrey GA, Saenger W (1991). Hydrogen Bonding in Biological Structures.

[14] Brock CP, Duncan LL (1994). Anomalous Space-Group Frequencies for Monoalcohols C_*m*_ H_*n*_ OH. Chem Mater.

[15] Shalaby MA, Fronczek FR, Vargas D, Younathan ES (1994). Conformations and Structure Studies of Sugar Lactones. Part III. The Composition and Conformation of D-Mannurono-γ-lactone in Solution, and the Structural Analysis of its β Anomer in the Solid State. Carbohydrate Res.

[16] Mueller U (1980). Strukturverwandtschaften unter den *E* Ph4+-Salzen. Acta Cryst.

[b17-j3prat] 17C. P. Brock and M. A. Lloyd, to be submitted to Section B of Acta. Cryst.

[18] Gerkin RE, Reppart WJ (1984). The Structures of the Lanthanide Ethyl Sulfate Enneahydrates *M*(C_2_H_5_SO_4_)_3_·9H_2_O [*M*=La–Lu (except Pm)], at 171 K. Acta Cryst.

